# Surface-Related Features and Virulence Among *Acinetobacter baumannii* Clinical Isolates Belonging to International Clones I and II

**DOI:** 10.3389/fmicb.2018.03116

**Published:** 2019-01-08

**Authors:** Jūratė Skerniškytė, Renatas Krasauskas, Christine Péchoux, Saulius Kulakauskas, Julija Armalytė, Edita Sužiedėlienė

**Affiliations:** ^1^Institute of Biosciences, Life Sciences Center, Vilnius University, Vilnius, Lithuania; ^2^INRA, UMR 1313 GABI, Plate-forme MIMA2, Jouy-en-Josas, France; ^3^INRA, MICALIS Institute, AgroParisTech, Université Paris-Saclay, Jouy-en-Josas, France

**Keywords:** *Acinetobacter baumannii*, clonal lineages, surface-related features, virulence, hydrophobicity

## Abstract

*Acinetobacter baumannii* currently represents one of the most important nosocomial infection agent due to its multidrug-resistance and a propensity for the epidemic spread. The *A. baumannii* strains belonging to the international clonal lineages I (IC I) and II (IC II) are associated with the hospital outbreaks and a high virulence. However, the intra and inter lineage-specific features of strains belonging to these most worldwide spread *A. baumannii* clones are not thoroughly explored. In this study we have investigated a set of cell surface-related features of *A. baumannii* IC I (*n* = 20) and IC II (*n* = 16) lineage strains, representing 30 distinct pulsed-field gel electrophoresis types in the collection of clinical isolates obtained in Lithuanian tertiary care hospitals. We show that *A. baumannii* IC II strains are non-motile, do not form pellicle and display distinct capsular polysaccharide profile compared with the IC I strains. Moreover, in contrast to the overall highly hydrophobic IC I strains, IC II strains showed a greater variation in cell surface hydrophobicity. Within the IC II lineage, hydrophilic strains demonstrated reduced ability to form biofilm and adhere to the abiotic surfaces, also possessed twofold thicker cell wall and exhibited higher resistance to desiccation. Furthermore, these strains showed increased adherence to the lung epithelial cells and were more virulent in nematode and mouse infection model compared with the hydrophobic IC II strains. According to the polymerase chain reaction-based locus-typing, the reduction in hydrophobicity of IC II strains was not capsule or lipooligosaccharide locus type-dependent. Hence, this study shows that the most widespread *A. baumannii* clonal lineages I and II markedly differ in the series of cell surface-related phenotypes including the considerable phenotypic diversification of IC II strains at the intra-lineage level. These findings suggest that the genotypically related *A. baumannii* strains might evolve the features which could provide an advantage at the specific conditions outside or within the host.

## Introduction

Gram-negative bacterium *Acinetobacter baumannii* is a difficult to treat infection agent, causing nosocomial infections worldwide (Eliopoulos et al., 2008; Holt et al., 2016). Characteristic features of this opportunistic pathogen include multidrug-resistance (MDR) phenotype, ability to withstand unfavorable environmental conditions for long periods of time and a high propensity for spread resulting in the hospital outbreaks, especially in the intensive care units (Manchanda et al., 2010; Eijkelkamp et al., 2014).

The worldwide spread of *A. baumannii* in clinical settings is characterized by the expansion of several predominant clones (Karah et al., 2012; Zarrilli et al., 2013). Of them, the international clonal lineages I (IC I) and II (IC II), otherwise known as European clones I and II, account for the most part of the *A. baumannii* infections (Zarrilli et al., 2013; Dahdouh et al., 2017). These clonal lineages have been identified around 1970s (Holt et al., 2016) and since then have been spread globally (Antunes et al., 2014). In particular, the IC II lineage strains are characterized through their high carbapenem-resistance and nosocomial spread in many countries during the recent years (Kim et al., 2017; Pournaras et al., 2017). The specific features contributing to the endemic nature of successful *A. baumannii* clones are of particular interest in exploring the virulome of *A. baumannii* (Di Nocera et al., 2011; Eijkelkamp et al., 2014; Ali et al., 2017) and identifying the novel candidates for the vaccine-based strategies to combat this infection agent (Ni et al., 2017). Most of the studies exploring virulent properties of *A. baumannii* are based on the experiments using several well-defined strains (Geisinger and Isberg, 2015; Tipton et al., 2015; Kentache et al., 2017). The data describing currently clinically relevant isolates are in scarce, despite findings that *A. baumannii* is characterized by its ability to change pathogenic features constantly (Antunes et al., 2014; Holt et al., 2016; Piepenbrink et al., 2016).

The cell surface structures are crucial for bacterial pathogens in sensing the environment and interacting with the host (Weber et al., 2016; Lee et al., 2017). In order to persist in clinical settings *A. baumannii* must be equipped with a set of cell surface features enabling it to adhere to the abiotic surfaces such metal and plastic found in medical devices and hospital equipment as well as to survive under desiccation stress (McConnell et al., 2013; Chiang et al., 2017). Multiple environmental and virulence-associated signals induce either biofilm formation or bacterial motility phenotypes through surface exposed sensors in *A. baumannii* (McConnell et al., 2013) assigning these features as possible survival-related factors. Moreover, specific cell surface determinants are required for *A. baumannii* to resist the host defense systems such as complement and macrophage-mediated killing as well as to mediate the attachment to the host cells at the sites of infection (Russo et al., 2010; Smani et al., 2012; Harding et al., 2018b).

In this study we present comprehensive comparative analysis of surface-related features and their relationship with *A. baumannii* IC I and IC II isolates and demonstrate that genotypically related strains display considerable cell-surface-related phenotypic differences that significantly impact their virulence properties displayed outside and within the host.

## Materials and Methods

### Description of Bacterial Strains and Growth Conditions

The 36 *A. baumannii* strains representing different pulsed-field gel electrophoresis (PFGE) defined pulsotypes from the retrospective collection of 365 clinical isolates previously genotyped by Povilonis et al. (2013) were used in the study (Supplementary Table S1). The strains were previously assigned to the international clonal lineages I and II using trilocus sequence-based typing (3LST). On the basis of MLST-IP typing, the IC I and IC II strains were assigned to ST1 and ST2, respectively (Povilonis et al., 2013). Strains were named by their clonal dependence. Roman numerals I and II indicate IC I and IC II, respectively, and lowercase letters represent different pulsotypes. The MLST typing of selected isolates using the Oxford scheme was undertaken according to *A. baumannii* MLST website^1^. Strains were cultured on the Luria-Bertani (LB) agar plates at 37°C. Liquid cultures were inoculated in LB medium and grown overnight.

### Determination of Cell Surface Hydrophobicity

Determination of cell surface hydrophobicity by salt aggregation test (SAT) was carried out as described by Nwanyanwu and Abu (2013). Briefly, *A. baumannii* were grown on LB plates at 37°C overnight. Cells were suspended in ddH_2_O until slight turbidity and mixed with the equal volume of ammonium sulfate solution to yield concentrations ranging from 0.0625 to 2 M. Cell aggregation (clumping) was observed under the microscope at 50x magnification. The cell surface hydrophobicity was expressed as a lowest salt concentration, which caused bacterial cell aggregation.

### Motility Assays

Twitching and swarming motilities were investigated as previously described (Eijkelkamp et al., 2011) with some modifications. Briefly, a single bacterial colony grown overnight, was collected using a sterile toothpick and stabbed through a semi-solid Tryptic Soy Broth medium (TSB) to the bottom of the Petri dish. For twitching and swarming motility assays, TSB medium with 0.75 and 0.25% agarose was used, respectively. Inoculated plates were kept in humid airtight containers and grown at 37°C for 24 or 48 h for surface motility and twitching motility, respectively. Motility was quantified by measuring the halo of growth around the inoculation site and expressed in millimeters.

### Assessment of Pellicle Formation

The pellicle formation was measured as described by Giles et al. (2015) with some modifications. The 12-well plates [tissue culture plates (TPPs)] with 3 ml of TSB medium were inoculated with 1000-fold dilutions of overnight bacterial cultures and incubated without shaking at 30°C for 30 h. To remove the pellicle from the surface of the medium, 200 μL of isopropanol was added to each well. The floating pellicle was removed and dissolved in 0.5 ml of 10 mM of NaOH immediately followed by the neutralization with HCl. The OD_600_ of the suspension was measured.

### Desiccation Assay

*Acinetobacter baumannii* were grown in LB medium at 37°C overnight and diluted into the same medium to the OD_600_ of 0.1. 10 μl of each of the sample were spotted on polystyrene, allowed to dry and incubated at 28°C for 24 h. Pre-desiccated samples were serially diluted by 10-fold dilutions and seeded on the LB plates. After desiccation, samples were resuspended in LB broth, serially diluted into 10-fold dilutions and seeded. LB plates were grown at 37°C overnight. Percentage of survived bacteria (D% – desiccation rate) was assessed by comparing obtained CFU counts of post-desiccated samples with those of the pre-desiccated samples.

### Biofilm Formation Assay

Experiments were carried out in LB medium. Overnight bacterial cultures were diluted 1000-fold, 200 μl of suspension was inoculated into the wells of 96 U-form polystyrene plate (Nerbe Plus) and incubated in static conditions at 37°C for 18 h. OD_600_ of planktonic culture was measured. The wells were then washed three times with 0.9% NaCl to remove non-adherent bacteria. Biofilms were stained with 0.5% crystal violet dye for 5 min and washed three times with 0.9% NaCl. Dye was eluted with 96% ethanol by incubation for 5 min, and OD_580_ was determined. The OD_580/600_ ratio was estimated to normalize the amount of formed biofilm to the total cell content.

### Adhesion to Polystyrene Assay

The adhesion tests were performed by dispensing 200 μL of x5 diluted overnight bacterial culture grown in LB medium into the wells of 96 F-form polystyrene plate (Nerbe Plus) and incubated for 2 h at 28°C. Wells were rinsed three times with PBS and stained with crystal violet as described above. The OD_580/600_ ratio was estimated to normalize the amount of adhered cells to the total cell content.

### Cell Culture Assays

Mouse epithelial LL/2 (LLC1) and mouse macrophages J774 cell lines were grown in Dulbecco’s modified Eagle’s medium (DMEM) (Gibco, 31966021) supplemented with 10% fetal bovine serum (FBS) (Gibco, 12657029) at 37°C with 5% CO_2_.

For adhesion experiment, lung epithelial cells were plated at a density of 1.5 × 10^4^ cells/well into 96-well TPP. Cells were grown for 48 h to form fastened culture monolayer with ∼80% confluence.

*A. baumannii* strains were cultured for 12 h until the log phase in LB medium at 37°C with moderate shaking at 145 rpm. All bacterial suspensions were equalized to yield OD_600_ = 0.7, washed once with PBS. LL/2 cells were infected with bacteria at a multiplicity of infection (MOI, bacteria: eukaryotic cell ratio) ∼1000:1. The number of *A. baumannii* CFUs inoculated per well was determined by serial dilution of bacterial culture in PBS and plating on the LB medium.

Infected LL/2 cell monolayers were incubated for 90 min at 37°C. Wells were carefully washed with DPBS three times to remove unattached bacteria. Then LL/2 cells were lysed with ddH_2_O by intense pipetting. Serially diluted lysates were plated onto LB medium to determine the number of adhered bacteria. Bacterial adherence (A%) to the LL/2 cells was expressed as a percentage of the CFU of adhered bacteria compared to the total number of CFUs of the initial inoculum.

### Phagocytosis Assay

For the phagocytosis assay, macrophages J774 were plated at a density of 5.6 × 10^4^ cells/well into 96-well TPPs and were grown for 14 h at 37°C with 5% CO_2_. Macrophages were infected with bacteria at MOI ∼200:1. Infected macrophages were then incubated for 60 min, washed three times in PBS and incubated with DMEM supplemented with 400 μg/μl of gentamycin for 30 min. After three washes with PBS, macrophages were resuspended in ddH_2_O and lysed by the intense pipetting. Serial dilutions were plated on the LB plates. Plates were incubated at 37°C overnight. Bacterial phagocytosis (P%) was expressed as a percentage of the CFU of intracellular bacteria compared to the total number of CFUs of the initial inoculum.

### *A. baumannii* Growth Assays

Bacterial growth was evaluated in LB medium, heat inactivated FBS (htFBS) and active FBS. FBS was inactivated by incubation at 56°C for 30 min with the constant shaking. Overnight cultures were inoculated at x1000 dilution to LB medium and 80% FBS or htFBS (20% of LB medium). Growth curves were measured at 37°C with periodic shacking every 20 min by Tecan Infinite M200 Pro microplate reader.

### Polymerase Chain Reaction

For the determination of capsule and lipooligosaccaharide (LOS) outer core (OC) locus types, a conventional polymerase chain reaction (PCR) was undertaken using primers listed in Supplementary Table S2. Conventional PCR volume was 20 μl and consisted of 2 μl of 10x Taq DNA polymerase reaction buffer with (NH_4_)_2_SO_4_ (Thermo Fisher Scientific), 2 mM MgCl_2_, 0.4 mM of each dNTPs, 400 nM of primers, 0.2 U Taq DNA polymerase (Thermo Fisher Scientific) and 1 μl of DNA template. Tm was calculated based on the primers sequences.

### Generation of Capsule Negative (Δ*wza*) *A. baumannii* Mutant

Markerless gene deletion was performed as previously described (Oh et al., 2015). Briefly, upstream and downstream regions of *A. baumannii wza* gene were amplified using primers listed in Supplementary Table S2 and DNA of IC II strain III-a as a template. The amplicons were joined with gentamicin resistance cassette *aac3I* amplified from a clinical *A. baumannii* strain by overlap PCR. The resulting DNA was cloned into pUC19_sacB plasmid yielding pUC19_sacB_UDwzaGm plasmid (Supplementary Table S2). IC II III-a strain was transformed with the resulting plasmid by electroporation and colonies were selected on LB agar with gentamicin. Colonies were picked up with sterile toothpick and inoculated in LB medium for 4 h. Serial dilutions were plated on LB agar with 10% sucrose. Mutants were identified by PCR with specific primers and confirmed by sequencing.

### Fractionation of Capsular Polysaccharides

For capsular polysaccharides (CPS) analysis, extracts of CPS from cell culture supernatants were prepared according to Geisinger and Isberg (2015). Briefly, cultures were grown on LB plates at 37°C overnight, suspended in PBS and normalized to an OD_600_ = 3. Polysaccharides were released into the supernatant by vortexing at maximum speed for 30 s. After centrifugation at 9000 × *g* for 10 min, polysaccharides were precipitated in 75% ice-cold ethanol overnight, followed by pelleting and air-drying. The pellet was resuspended in SDS sample buffer and boiled for 5 min. Samples were loaded on the 12% SDS-PAGE gels. After electrophoresis, gels were stained overnight with 0.1% (w/v) Alcian Blue as described in Mercaldi et al. (2008).

### Transmission Electron Microscopy

Transmission electron microscopy (TEM) analysis was undertaken at Microscopy and Imaging Platform MIMA2 at Gabi UMR (Jouy-en-Josas, France). Bacteria were grown in LB medium at 37°C overnight and cells were fixed within 0.1 M sodium cacodylate buffer (pH 7.2) with 2% of glutaraldehyde for 3 h at room temperature. After treatment with 0.5% Oolong Tea Extract (OTE) in cacodylate buffer, post-fixation with 1% osmium tetroxide containing 1.5% potassium cyanoferrate, pellets were dehydrated in solutions of increasing ethanol concentrations and embedded in Epon. Ultrathin sections were collected on 200-mesh copper grids and counterstained with lead citrate. Grids were examined with a Hitachi HT7700 electron microscope operated at 80 kV (Elexience, France), and images were acquired with a charge-coupled device camera (AMT).

### *Caenorhabditis elegans* Fertility Assay

*Caenorhabditis elegans* N2 eggs were grown to stage L1 and arrested overnight at 20°C to physiologically synchronize the worms. Nematodes were grown on nematode growth medium plates (NGM) with the culture of *Escherichia coli* OP50 strain until nematodes reached L2 stage. Overnight cultures of different *A. baumannii* strains were seeded on NGM medium. Ethanol was added to a final concentration of 1%, as it was demonstrated that ethanol induces *A. baumanni* virulence (Smith et al., 2007), and plates were grown at 21.5°C for 24 h. One L2 stage worm was placed over each *A. baumannii* strain and incubated at 21.5°C. On the third day after infection worm progeny was determined by counting *C. elegans* worms.

### Sepsis Model in Mice

Eight- to twelve-week-old female BALB/c mice were purchased from Institute of Biochemistry, Life Science Center (Vilnius University, Vilnius). The animals were maintained and used in accordance with the recommendations of the directive 2010/63/EU of the European Parliament and of the Council of 22 September, 2010 on the protection of animals used for scientific purpose. Study was performed under permission of Lithuanian State Food and Veterinary Service No. G2-72.

A sepsis model was established as described previously (Huang et al., 2014). Briefly, *A. baumannii* cultures were grown in LB medium for 18 h at 37°C and adjusted to the designated concentrations with PBS according to the OD_600_ values based on previously determined concentrations by seeding serial dilutions and counting CFUs. Samples were prepared by mixing the bacterial suspension with 5% of porcine mucin (w/v; Sigma-Aldrich). Mice were injected intraperitoneally with 0.5 mL of the sample. CFUs corresponding the bacterial loads were determined by plating sequential dilutions on LB plates. Bacterial colonies obtained from animal sources were confirmed by PCR with *A. baumannii*-specific primers (Supplementary Table S2).

### Statistical Analysis

All statistical comparisons were based on the one-way analysis of variance (ANOVA) with a Tukey HSD *post hoc* test. Statistical analysis was performed using the computing environment R version 3.5.1 (R Development Core Team, 2008). All quantitative data are representative of at least three independent experiments.

## Results

### *A. baumannii* IC II Strains Are Non-motile and Pellicle-Non-Forming Compared With the IC I Strains

Thirty six *A. baumannii* clinical isolates, chosen for the present study, were representatives of 30 distinct PFGE types of IC I and IC II clonal lineages, and were obtained from Lithuanian hospitals during the period of June–November 2010 and characterized in a previous study (Povilonis et al., 2013). Twenty IC I isolates and 16 IC II isolates were selected (Supplementary Table S1). All isolates were multidrug-resistant (resistant to three or more antibiotic classes).

**FIGURE 1:**
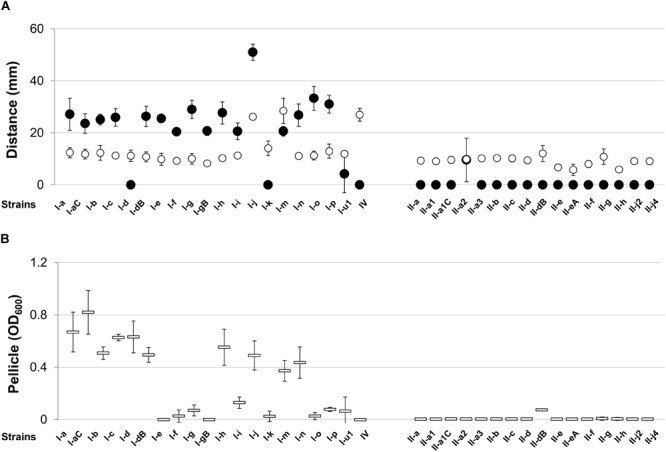
Twitching and swarming motilities and pellicle formation of *Acinetobacter baumannii* IC I and IC II lineage strains. **(A)** Twitching (●) and swarming (o) motilities expressed as a distance in mm; **(B)** total pellicle biomass (=) was suspended in the aqueous solution and absorbance OD_600_ was measured. Data are given as mean ± standard deviations from three independent experiments. Roman numerals I and II in the strains names indicate IC I and IC II, respectively.

We were interested, whether representative strains of the two most common clonal lineages display specific pattern of surface-related features, which are thought to be important for *A. baumannii* growth and survival in clinical environment and within the host (Rumbo et al., 2014; Weber et al., 2016; Lee et al., 2017). Therefore, we first tested swarming and twitching motility of selected *A. baumannii* isolates. The swarming distance, expressed by the majority of strains (92%, 33/36), was low and yielded approximately 6–14 mm. Only three strains, all representatives of IC I lineage, showed increased swarming motility yielding a value of >26 mm (Figure 1A). However, the majority of *A. baumannii* IC I lineage strains showed twitching motility in contrast to IC II strains, which lacked this property with the exception of a single strain (Figure 1A). The IV type pili have been proposed to be responsible for twitching motility in *A. baumannii* (Harding et al., 2013), therefore we looked for the presence of pili-like structures on the cell surface. The transmission electron microscopy of representative motile IC I strain 169 and non-motile IC II strain II-a showed marked differences in cell surface structures, the IC I strain displaying pili-like extended structures, which were absent in the IC II strain (Supplementary Figure S1).

According to the recent observations, the bacterial motility contributes to the formation of pellicle, a biofilm at the air–liquid interface (Giles et al., 2015; Hölscher et al., 2015). Hence, we tested the ability of our set of *A. baumannii* strains to form pellicle by growing them in TSB medium as described in Section “Materials and Methods”. The vast majority of IC II lineage strains lacked the ability to form pellicle, whereas 85% (17/20) of IC I strains showed pellicle-forming phenotype (Figure 1B). The pellicle formation clearly was a trait of IC I lineage, though these strains were highly various in terms of the abundance of pellicle biomass. However, there was no obvious correlation among pellicle formation and swarming or twitching motility in IC I group, as for example I-d strain lacking twitching motility phenotype, was able to form a pellicle and I-gB strain with no pellicle forming feature was able to demonstrate twitching motility.

Therefore, results show evident differences in twitching motility and pellicle formation phenotypes between *A. baumannii* IC I and IC II lineage strains.

### *A. baumannii* IC II Strains Express Different Type of Capsular Polysaccharide Profiles Compared With IC I Strains

**FIGURE 2:**
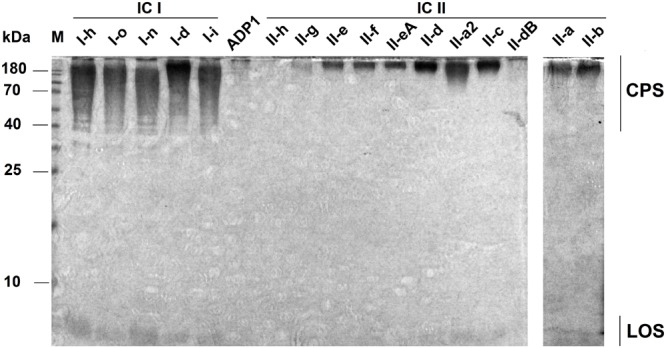
Capsular polysaccharide (CPS) profiles of *A. baumannii* IC I and IC II lineage strains. 12% SDS-PAGE followed by Alcian Blue staining was undertaken to visualize CPS. *Acinetobacter baylyi* strain ADP1 was used as CPS-negative control. LOS denotes lipooligosaccharide (LOS). The positions of standard molecular mass markers [PageRuler^TM^ Prestained Protein Ladder (Thermo Fisher Scientific)] are shown on the left.

Since exopolysaccharide represents one of the bacterial pellicle components (Armitano et al., 2014; Nait Chabane et al., 2014) and given the fact that IC I and IC II lineage strains exhibited marked differences in the pellicle formation, we next asked whether they differ in a production of cell surface glycoconjugates such as CPSs and lipooligosaccharide (LOS). Figure 2 shows polysaccharide profiles of representative IC I (*n* = 5) and IC II (*n* = 11) isolates from our tested set of 36 isolates (profiles of the rest of isolates are given in the Supplementary Figure S2). Major differences can be observed in CPS profiles between representatives of two clonal lineages. The IC I strains express CPS of variable length, whereas the IC II strains with the few exceptions, produce only narrow distribution, high-molecular-weight CPS (Supplementary Table S1). Two IC II strains (II-h and II-dB) were found to be capsule-deficient at growth conditions used (Figure 2).

Therefore, these observations imply that *A. baumannii* belonging to the most widespread clonal lineages display lineage-specific CPS composition.

### *A. baumannii* IC II Strains Exhibit Variation in the Surface Hydrophobicity Compared With IC I Strains

One of the most important characteristic of bacterial surface is cell surface hydrophobicity, whereas it plays a role in virulence-associated processes (Krasowska and Sigler, 2014). Since our tested strains differ in CPS production, we decided to access hydrophobicity of *A. baumannii* clinical isolates. We performed SAT, which is based on the clumping of bacteria in the presence of salt (Nwanyanwu and Abu, 2013).

**FIGURE 3:**
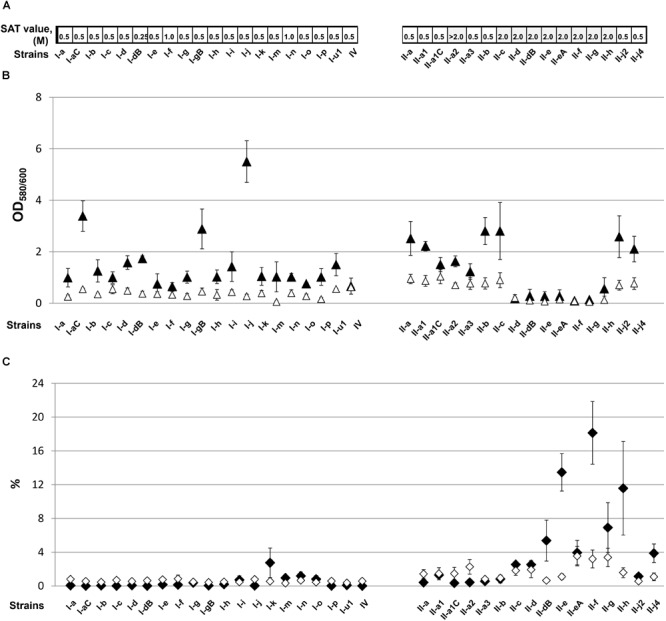
The cell surface-associated features of *A. baumannii* IC I and IC II lineage strains. **(A)** Surface hydrophobicity, defined by the salt aggregation test (SAT) and expressed as a minimum ammonium sulfate concentration (M) required for bacterial aggregation; **(B)** biofilm formation (▲) and adherence to polystyrene (Δ) expressed as OD_580/600_. Error bars represent standard deviations from three independent experiments; **(C)** desiccation resistance (♦) and adhesion to lung epithelium cells LL/2 (♢) expressed as percentages. Error bars represent standard errors from at least three independent experiments. Roman numerals I and II in the strains names indicate IC I and IC II, respectively.

All tested IC I strains were considered to have hydrophobic character based on the estimated SAT values, which ranged from 0.5 to 1 M (Figure 3A). In contrast, more than a half (56%, 9/16) of IC II strains displayed low surface hydrophobicity compared with the IC I group (SAT values ≥ 2 M) (Figure 3A). Of hydrophilic IC II strains, II-a2, II-c, II-d, and II-dB represented clonal isolates, with strains belonging to the pulsotypes retrieved repeatedly from the hospitals, whereas II-e, II-eA, II-f, II-g, II-h isolates were sporadic (Supplementary Table S1). We did not observe a correlation between hydrophobicity and sequence type (ST) by examining selected IC II isolates according the Oxford multilocus sequence typing (MLST) scheme^1^. Thus, strains II-a and II-f, differing significantly in hydrophobic features, were both assigned to a common sequence type ST208, whereas strains II-a2 and II-h displaying hydrophilic character were assigned to different STs, ST 440 and ST348, respectively.

Notably, the majority of the hydrophilic IC II strains showed significantly reduced growth rates in the LB medium compared with hydrophobic IC II strains, indicating altered bacterial fitness of hydrophilic strains (Supplementary Figure S3).

### Hydrophilic *A. baumannii* IC II Strains Lack the Capacity to Form Biofilms and Adhere to the Plastic Surface

Since, it has been proposed that a degree of cell surface hydrophobicity could modulate adhesive properties of various commensal and pathogenic microorganisms (Krasowska and Sigler, 2014), we investigated how hydrophobic character of *A. baumannii* IC I and IC II strains impacts their ability to form biofilms and adhere to the abiotic and biotic surfaces. Interestingly, the trend of biofilm formation among IC I and IC II strains was different to that observed for pellicle phenotype (Figure 3B). All IC I strains and some IC II strains formed biofilm, albeit at the varying levels, whereas a group of IC II strains that were genetically close according to the PFGE analysis (Povilonis et al., 2013), namely, II-d, II-dB, II-e, II-eA, II-f, II-g, and II-h showed extremely weak biofilm-forming ability or entirely lacked this phenotype (Figure 3B). Biofilm non-forming phenotype of these isolates correlated with their low surface hydrophobicity according to the SAT assay.

Current model of biofilm formation involves attachment phase when bacteria comes into contact with the surface (Krasowska and Sigler, 2014). Due to the fact that different *A. baumannii* strains possess various surface hydrophobicity it could result in differences in the initial attachment. Therefore *A. baumannii* adherence to plastic was assessed. As can be seen in Figure 3B, all tested IC I lineage strains, except two (I-m and I-p), exhibited modest adherence to polystyrene. Again, IC II strains with the low cell surface hydrophobicity and impaired biofilm-forming phenotype, poorly adhered to the plastic, indicating that hydrophobicity in *A. baumannii* is essential in the initial attachment to the surface (Figure 3B).

Taken together, these results demonstrate that a high cell surface hydrophobicity of *A. baumannii* impacts important virulence traits such as biofilm formation and adherence to the abiotic surface.

### Hydrophilic *A. baumannii* IC II Strains Survive Desiccation Stress Better Compared With Hydrophobic IC II Strains

Observation that some *A. baumannii* clinical strains exhibited low cell surface hydrophobicity, prompted as to evaluate how it impacts the ability to survive desiccation stress, feature which contribute to bacterial survival and persistence in a hospital environment (Russotto et al., 2015).

**FIGURE 4:**
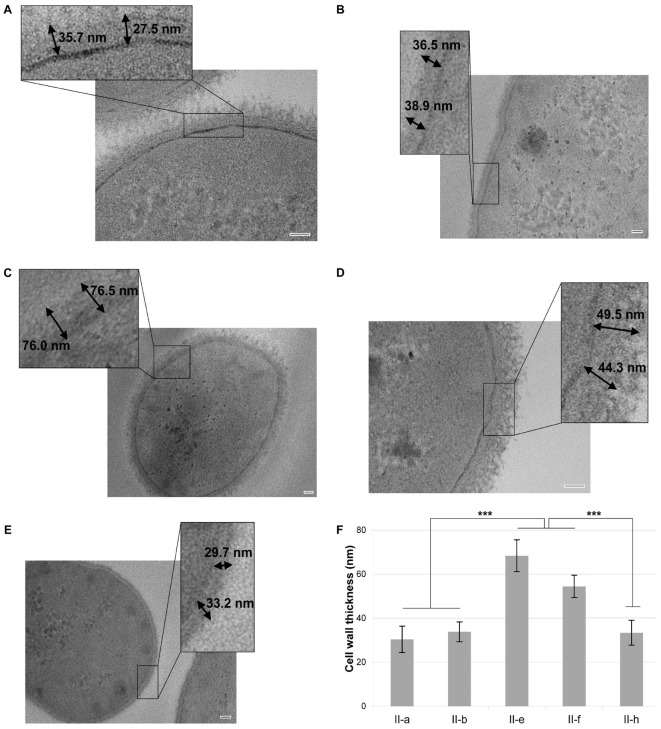
Cell wall thickness of *A. baumannii* strains with different surface hydrophobicity, assessed by the transmission electron microscopy. *A. baumannii* II-b **(A)**, II-a **(B)**, II-f **(C)**, II-e **(D)**, and II-h **(E)** strains were analyzed. Scale bar is 50 nm. Double arrows in the larger scale insets show the calculated thickness of the cell walls. **(F)** Average of cell wall thickness of the strains. Error bars represent standard deviations from three measurements of three different cells, significance was assessed by ANOVA. ^∗∗∗^*p* < 0.001.

Overall, the majority of IC I and a part of IC II strains poorly survived desiccation, yielding only 0.005 to 1.3% of survived cells (Figure 3C). However, a group of hydrophilic IC II strains was highly resistant to desiccation and displayed 2–14 times higher resistance compared with the rest of the IC II strains (Figure 3C). The decreased surface hydrophobicity possibly might increase water retention in the bacterial cell wall and thus contribute to the desiccation resistance. Moreover, we observed that the hydrophilic IC II strains II-e and II-f, displaying the highest desiccation resistance among tested strains, had approximately twofold thicker cell wall compared with the hydrophobic strains II-a and II-b and a capsule-deficient strain II-h according to the TEM analysis (Figures 4A–F).

Therefore, the hydrophilic nature of cell surface strongly impacts *A. baumannii* ability to resist desiccation.

### Hydrophilic *A. baumannii* IC II Strains Adhere Better to the Lung Epithelial Cells Compared With the Hydrophobic IC II Strains

*A. baumannii* adherence to the epithelial cells is thought to be one of the essential features required for infection process (Smani et al., 2012). Therefore, we hypothesized that a degree of cell surface hydrophobicity might play a role in *A. baumannii* adherence to the host cells as well. Overall, the specific adherence of *A. baumannii* clinical strains to the mouse lung epithelial LL/2 cells was poor (Figure 3C). The IC I strains adhered at a rate of 0.3 to 0.8%, while the adhesion of IC II strains was more pronounced and yielded 0.5 to 3.5% rate. In the IC II group of hydrophilic strains, in particular II-a2, II-c, II-d, II-eA, II-f, II-g, and II-h strains showed a clear trend of increased ability to adhere to the epithelial cells (Figure 3C).

Therefore, the results presented above imply, that IC I and IC II strains might use different cell surface-related properties for an attachment to the host cells. Moreover, our observations with IC II strains also suggest that genetically related *A. baumannii* strains belonging to the same clonal lineage, display a marked variation in the surface-related properties. Therefore, we hypothesized that these phenotypic differences could have different impact on their virulence properties and decided to investigate them more thoroughly.

### The Presence of *A. baumannii* Capsule Is Essential for the Resistance to Macrophage Phagocytosis and Serum-Mediated Killing

**FIGURE 5:**
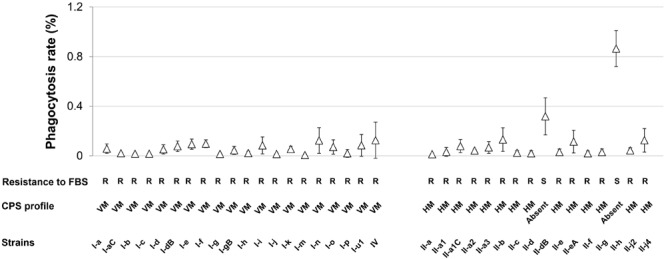
Susceptibility of *A. baumannii* IC I and IC II lineage strains to macrophage and serum-mediated killing. Phagocytosis by J774 macrophages is expressed as percentages and shown in a graph (Δ). Error bars represent standard deviations from three independent experiments. R and S letters below the graph denote the strains that are FBS resistant and sensitive, respectively. HM and VM indicate profile of high and variable-molecular-mass CPS, respectively. Roman numerals I and II in the strains names indicate IC I and IC II, respectively.

As the evasion of host defense systems such as phagocytic cells and complement are crucial in *A. baumannii* infection (Russo et al., 2010), we characterized our set of IC I and IC II isolates by the rate of macrophage-mediated phagocytosis and by the ability to survive serum-mediated killing. No correlation between strains clonal dependence or surface hydrophobicity and resistance to macrophage or serum killing was observed. All tested strains exhibited low rate of macrophage-mediated phagocytosis ranging from 0.01 to 0.18% with the exception of strains II-dB and II-h, which demonstrated approximately 5 and 10 times higher phagocytosis rate, respectively (Figure 5 and Supplementary Table S2). Furthermore, these two strains showed reduced growth in serum-supplemented LB medium compared with the rest of the strains, which were considered resistant to complement system (R) (Figure 5 and Supplementary Table S2). Notably, II-dB and II-h strains were the only ones CPS-deficient according the SDS-PAGE assay (Figure 2), indicating the crucial role of *A. baumannii* capsule in the evasion of host defense system. The role of capsule was further supported by the analysis of *A. baumannii* IC II strain III-a deletion mutant of *wza* gene, which is essential (Russo et al., 2010) for capsule synthesis. The *Δwza* strain showed drastic increase in the FBS-sensitivity and displayed an impairment of CPS synthesis compared with the parent strain (Supplementary Figure S4).

**FIGURE 6:**
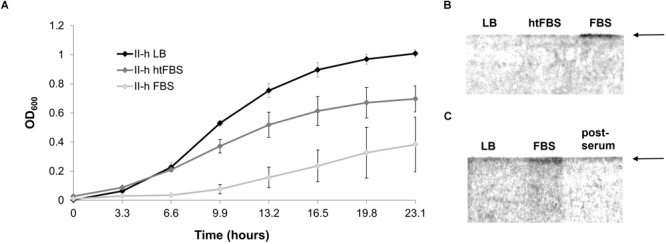
Growth and CPS production by *A. baumannii* IC II strain II-h. **(A)** Growth curves in LB, LB supplemented with 80% of heat treated (htFBS) and untreated FBS. Error bars represent standard deviations from three independent experiments; **(B)** SDS-PAGE gel showing the polysaccharide profiles from cells cultured for ∼24 h in LB, LB supplemented with 80% of heat treated and untreated FBS; **(C)** SDS-PAGE gel showing the polysaccharide profiles from the cells cultured for 24 h in LB with FBS, then re-cultured for 24 h in fresh LB without FBS (post-serum). Gels were stained with Alcian Blue. Arrows on the right indicate the position of CPS.

Strikingly, the serum sensitive strain II-h was able to restore growth in the serum-containing LB medium after culturing for approximately 10 h (Figure 6A). Therefore, we asked, whether this restoration is due to the induction of CPS synthesis. Indeed, the results of SDS-PAGE indicated the presence of a band corresponding CPS, which was absent in the same strain cultured in the LB medium or in the same medium supplemented with the heat inactivated serum (Figure 6B). Production of CPS was lost as soon as serum-induced bacteria were repeatedly re-grown in LB media (Figure 6C).

Thus, the results demonstrate that the capsule is essential for *A. baumannii* resistance to macrophages and serum-mediated killing and that capsule production might be induced by the presence of serum.

### Hydrophilic *A. baumannii* IC II Strains Tend to Exhibit Enhanced Virulence in *C. elegans* and Murine Infection Model Compared With Hydrophobic IC II Strains

Next, we investigated virulence of *A. baumannii* using nematodes fertility assay and mouse sepsis infection model. First, to assess the virulence in *C. elegans*, we have selected a set of IC II strains, displaying different properties of surface hydrophobicity, resistance to desiccation and ability to adhere to the lung epithelial cells. The strains II-b and II-a are hydrophobic, show poor resistance to desiccation and have a weak capacity to adhere to the epithelial cells, whereas strains II-f, II-e, and II-h are hydrophilic, highly resistant to desiccation and show cell adherence ability (Supplementary Table S1). All selected strains except II-h express CPS under laboratory growth conditions (Figure 2).

**FIGURE 7:**
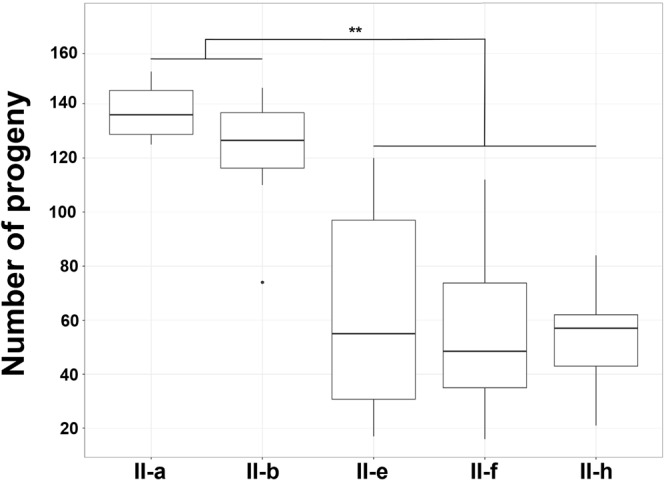
*Caenorhabditis elegans* fertility assay. Box plot of the count of nematodes progeny after 3 days of incubation in the presence of *A. baumannii* IC II lineage strains exhibiting different cell surface hydrophobicity. Data are from three independent experiments, three to four plates were used in the each experiment. Black lines represent medians and whiskers – minimum to maximum values, significance was assessed by ANOVA. ^∗∗^*p* < 0.01.

*Caenorhabditis elegans* fertility assay demonstrated that a total number of progeny after 3 days upon infection was approximately two times lower in nematodes infected with the hydrophilic strains II-f, II-e, and II-h compared to those infected with the hydrophobic strains II-a and II-b and the difference was statistically significant (*p* < 0.01) (Figure 7). Moreover, other hydrophilic strains of IC II lineage also displayed a trend of increased virulence in nematodes compared with the hydrophobic strains of the same lineage (Supplementary Figure S5). Notably, strain II-h, being capsule-deficient under laboratory conditions, showed similar virulence features in *C. elegans* compared with capsule-producing strains II-e and II-f.

**FIGURE 8:**
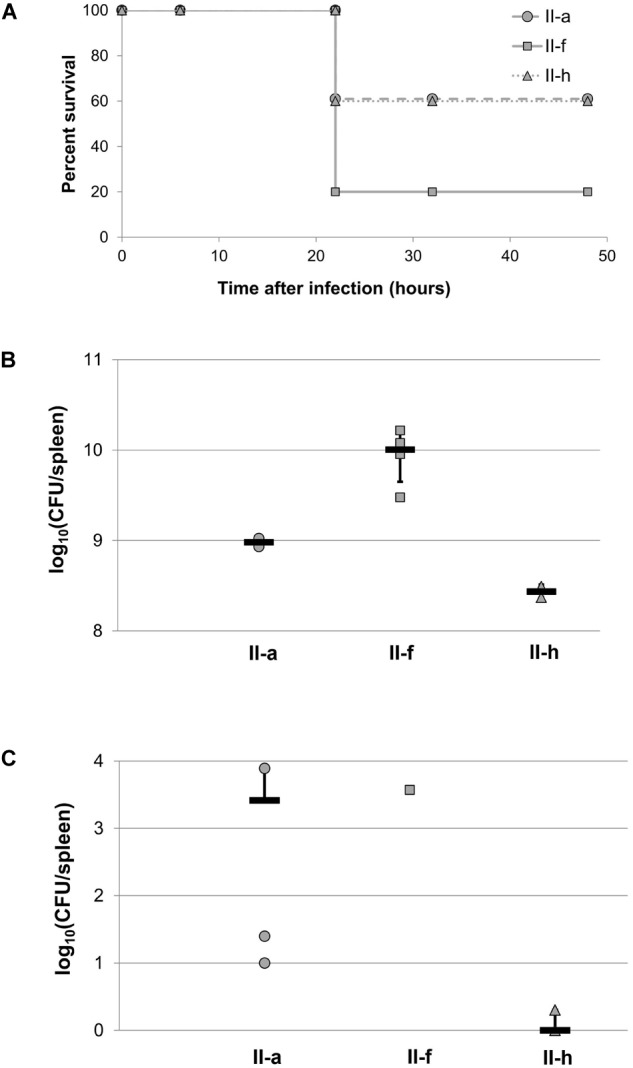
*Acinetobacter baumannii* sepsis infection in BALB/c mice. Mice (*n* = 5 per group) were intraperitoneally infected with 1.25 × 10^6^ CFU of II-a, 5.5 × 10^6^ CFU of II-f and 2.25 × 10^6^ CFU of II-h *A. baumannii* IC II lineage strains. **(A)** Survival of mice; **(B)** bacterial load, estimated in the spleen *post-mortem*; **(C)** bacterial load in the spleen after 48 h of infection. Survived mice were sacrificed and spleens were tested for bacterial loads. Black lines in **(B,C)** represent an average, error bars represent standard deviations.

Next, we used an experimental murine model to assess the ability of selected IC II strains to establish a systemic infection. Representative IC II strain II-a with hydrophobic cell surface properties, II-f strain with hydrophilic character, and hydrophilic II-h strain, albeit displaying capsule-non-producing phenotype were used for infection. The mice survival rates were monitored for several days. The mice infected with II-f strain showed twofold higher mortality rate compare to those infected with II-a and II-h strains (80% vs. 40%) (Figure 8A). Spleens from the mice, infected with the II-f strain and examined *post-mortem* had 10 times higher bacterial load compared with those from the mice infected with II-a strain, and over 30 times higher load compared with those infected with capsule-deficient II-h strain (Figure 8B). Furthermore, the higher yield of bacteria was detected in spleen from the mouse, which survived after 48 h upon inoculation with II-f strain, while in the case of II-a strain the load of survived bacteria was mainly lower and only a few II-h colonies were observed after mice sacrifice and spleens examination (Figure 8C).

Therefore, the low cell surface hydrophobicity clearly impacts virulence of *A. baumannii*, although our study predicts that the capsule’s presence is critically needed to establish an infection in vertebrate host, but not in *C. elegans* model.

### Variations in Cell Surface Hydrophobicity Among *A. baumannii* IC II Strains Are Not Dependent on the Types of Capsule or Lipooligosaccharide Synthesis Loci

Observations that the low cell surface hydrophobicity of *A. baumannii* IC II strains correlates with their enhanced virulence *in vivo* prompted as to investigate the cell surface determinants that might be responsible for this phenotype. Earlier, it was demonstrated that changes in the structure of cell surface components, such as lipopolisaccharides, could alter bacterial surface hydrophobicity and biofilm formation in *E. coli* (Nakao et al., 2012).

First, we were interested whether IC II strains displaying different degree of cell surface hydrophobicity posses differences in CPS synthesis locus (K locus) organization, as *A. baumannii* is known to produce a high diversity of CPS, which impacts cell surface properties (Traub and Bauer, 2000). For this purpose, we intended to determine the variants of Wzy polymerase gene present in the K locus according to the *A. baumannii* K locus typing scheme developed by Hu et al. (2013). Wzy polymerase is responsible for polymerization of oligosaccharide units into CPS (Kenyon and Hall, 2013). Hu et al. (2013) have demonstrated a strong correlation between the *wzy* gene variant and organization of CPS synthesis gene cluster. We have chosen the IC II strain II-f for the amplification of approximately 8 kb in length K locus region with primer pair gnaaF/galuR (Supplementary Table S2). Sequencing of the resulting amplicon revealed the presence of PSgc12 gene cluster with the *wzy11* gene variant, what is in accordance with the typing scheme proposed. The *wzy11* gene variant so far was found to occur in *A. baumannii* possessing KL2 capsule type (Kenyon et al., 2014a). To identify whether *wzy11* gene variant was present in other IC II strains, we have designed *wzy11*-specific primers wzy11F/wzy11R and used them for PCR with the DNA of IC II strains. All tested IC II strains, except II-a2, II-g and II-h, yielded amplicons specific to *wzy11* gene variant suggesting the presence of the same type of CPS synthesis locus in the majority of IC II strains regardless their hydrophobic features. We have designed additional primer pairs targeting other possible *wzy* gene variants and probed them with the rest of the strains (Supplementary Table S2). All IC I strains, except I-e and I-f, coded for the *wzy* variant found in *A. baumannii* with the KL40 capsule type. The I-e and I-f strains carried *wzy* allele, present in strains assigned to KL1 type, whereas the IC II II-a2 strain had *wzy* gene variant found in *A. baumannii* strain with the KL27 capsule type. The designed primers were not able to target the *wzy* gene in IC I strain IV and IC II strains II-g and II-h.

Next, we identified the type of LOS by using typing scheme developed by Kenyon et al. (2014b). Typing scheme is based on the determination of a locus, responsible for the synthesis of the OC component of the LOS in *A. baumannii*. The strains of IC I and IC II lineages have been previously shown to display variations in the OC component (Kenyon et al., 2014b). The PCR with the RH1704/RH1705 primer pair (Supplementary Table S2) and the DNA obtained from *A. baumannii* IC II strains resulted in an amplicon corresponding to the *gtrOC4*-*gtrOC5* region in the OC gene cluster and thereby confirming the OCL1 variant for all tested strains.

Therefore, typing of *A. baumannii* genome regions responsible for the synthesis of polysaccharides suggest that neither capsule synthesis locus type, nor OC locus type of LOS are responsible for variations in the surface hydrophobicity displayed by the *A. baumannii* IC II strains.

## Discussion

In this study we have performed a comprehensive investigation of the cell surface-related phenotypic properties of *A. baumannii* clinical strains belonging to the international clonal lineages IC I and IC II. We aimed to identify their relations with the virulence, since specific clone-associated features are largely obscure.

In accordance with other studies (Eijkelkamp et al., 2011), our results show that swarming motility is rare phenotype, as we have identified only three strains with this type of motility. While swarming motility in the most of bacteria is flagella-depended, bioinformatic analysis did not indicate any flagellar genes in *A. baumanni* genomes (Clemmer et al., 2011). However it was observed that some *A. baumannii* strains indeed are able to express swarming motility on the solid surfaces (Clemmer et al., 2011; Eijkelkamp et al., 2011; McQueary et al., 2012). It was suggested that swarming motility in *A. baumannii* is rather flagella-independent, multifactorial and complex process (Harding et al., 2013).

Our results indicate that twitching motility and pellicle formation are features strongly associated with *A. baumannii* IC I lineage strains. These data are in the line with the previous observations of Eijkelkamp et al. (2011) from the analysis of Australian *A. baumannii* clinical isolates, where all IC I clone and only a few IC II clone members showed twitching motility. *A. baumannii* twitching motility is flagella-independent phenomenon and has been shown to require type IV pili (Harding et al., 2013). Comparative genomic studies demonstrated that IC I and IC II isolates carry the same sets of essential genes responsible for type IV pili biogenesis, although possess lineage-specific differences in a soluble domain of the major pili subunit PilA (Piepenbrink et al., 2016) hypothesizing that it might render differences in twitching motility observed between the lineages. Moreover, the IC II genomes, in contrast to the IC I, carry the *tfpO* gene, coding for a pilin glycosylating *O*-oligosaccharyltransferase (*O*-OTase) in addition to PglL-like *O*-OTase present in all *Acinetobacter* species (Harding et al., 2015). Strikingly, the possession of *tfpO* gene correlated with the presence of the C-terminal serine residue in the PilA protein (Harding et al., 2015), suggesting the C-terminal glycosylation event, which may affect pilin-associated phenotypes. However, our generated *A. baumannii tfpO* gene deletion in IC II strain II-d had no effect on the twitching motility (data not shown) indicating that other components are responsible for this phenotype. This observation is supported by the analysis of C-terminal glycosylation-deficient PilA S136A mutant of *A. nosocomialis*, which showed no pili-associated phenotypic changes compared with the parent strain (Piepenbrink et al., 2016).

Similarly to other Gram-negative pathogens, *A. baumannii* CPS and lipopolysaccharide (LPS), the latter thought to be deficient in extracellular polysaccharide portion in most *A. baumannii* strains (Harding et al., 2015) and called LOS, are essential virulence factors protecting from the host complement system (Russo et al., 2010) and mediating inflammatory responses (Moffatt et al., 2013). Our data show that nearly all clinical *A. baumannii* strains produce capsule, although IC I and IC II strains display lineage-specific CPS profile. According to the previous reports, *A. baumannii* genomic K locus, responsible for the CPS synthesis and export, is highly diverse (Hu et al., 2013; Kenyon and Hall, 2013). Strikingly, all our examined capsule-producing IC I strains synthesized polysaccharides of variable-molecular-mass, whereas IC II strains yielded exclusively high-molecular-mass CPS. The data on *A. baumannii* CPS profiling are in scarce. According to the recent reports, *A. baumannii* non-clonal strain ATCC 17978 produced high-molecular-mass CPS (Lees-Miller et al., 2013; Geisinger and Isberg, 2015), whereas IC I strain AB307-0294 yielded CPS of variable-molecular-mass (Russo et al., 2016). Interestingly, Dams-Kozlowska and Kaplan (2007) found that the length of the polysaccharide produced by *Acinetobacter venetianus* strain Rag-1 depends on the introduction of the point mutations into the proline-glycine rich region of the Wzc protein, a member of Wzy-dependent polysaccharide production pathway (Hu et al., 2013). The Wzc tyrosine kinase belongs to the group of polysaccharide co-polymerases (PCPs), proposed to control the capsule polysaccharide length in Gram-negative bacteria (Kalynych et al., 2012). Wzc activity has been shown to be required for *A. baumannii* CPS assembly, since introduced mutations in the kinase Walker motifs and phosphorylation site resulted in the changes of CPS profile (Geisinger and Isberg, 2015). Recently, Harding et al. (2018a) found that introduction of a non-native ortholog of phosphoglycosyltransferase PglC resulted in a change of capsule serotype and CPS profile of *A. baumannii*, indicating that genetic alterations in other capsule-related genes might be involved in the capsule variations. We have performed phylogenetic analysis of Wzc and PglC proteins of *A. baumannii* IC I and IC II strains from the genomes available in the databases (Supplementary Figure S6). The sequences of Wzc and PglC proteins of non-clonal *A. baumannii* strain ATCC 17978 and *A. venetianus* strain Rag-1, for which CPS profiles have been determined, were also included. The IC II strains and non-clonal ATCC 17978 strain formed a separate cluster regarding both proteins, whereas *A. venetianus* strain Rag-1 tend to group with the IC I cluster. The observed clustering correlated with identified CSP profiles of IC II and IC I strains with the same capsule type as aligned *A. baumannii* strains from the databases as well as with reported CSP profiles for ATCC 17978 and *A. venetianus* strain Rag-1. This indicates that distinct CPS profiles could possibly be triggered by the genetic differences in capsule-related genes between two clones.

All our analyzed IC I and IC II strains showed low macrophage-mediated phagocytosis rate and high resistance to FBS, except for the capsule-deficient strains II-dB and II-h, thereby confirming the crucial role of capsule (Russo et al., 2010) in the evasion of host immunity components. Moreover, induction of capsule synthesis, demonstrated by strain II-h after prolonged incubation in the presence of FBS, indicates that capsule production might display a phase variation pattern, recently shown for capsule expression in *A. baumannii* strain AB5075 (Chin et al., 2018). Previously, *A. baumannii* CPS expression was also demonstrated to be depended on the presence of antibiotics, where hyperproduction of capsular exopolysaccharide was reversible and non-mutational (Geisinger and Isberg, 2015). Our results demonstrate that capsule-deficient strain II-h exhibited similar virulence in *C. elegans* infection model as did capsule-producing hydrophilic strains, while in a murine model its virulence was suppressed. The presence of the capsule might be crucial at the beginning of infection in vertebrates, when phagocytic cells and active complement proteins neutralize bacteria. The defense system in nematodes is based mostly on the antimicrobial peptides and lectins (Ermolaeva and Schumacher, 2014), there is no evidence for the presence of functional complement proteins or phagocytic cells. Russo et al. (2010) showed that capsule-negative *A. baumannii* mutant was unable to survive in the rat soft tissue infection model during the first hours after injection. However, in our study II-h strain was able to cause the death in murine model, possibly indicating ability to induce capsule production, reach infectious dose and expand the contagion.

Another feature that clearly distinguishes strains of IC I and IC II lineages is the inability of IC II strains to form pellicle in contrast to the most of IC I lineage members. Pellicle represents a biofilm formed by bacteria at the air–liquid interface (Armitano et al., 2014). It is composed of cells surrounded by complex extracellular matrix containing exopolysaccharides, LOSs, lipids, DNA and protein components (Nait Chabane et al., 2014). The plastic and metal surfaces might serve as a basis for the attachment of the formed pellicle (Nait Chabane et al., 2014; Giles et al., 2015), therefore medical devices containing the liquid represent niches for pellicle formation and *A. baumannii* colonization. The previous studies reported increased expression of virulence factors such as phospholipases, adhesion factors, type VI secretion system, siderophore iron uptake systems implies the role of *A. baumannii* pellicle in the virulence (Marti et al., 2011; Kentache et al., 2017). There is a controversy regarding observations on the impact of *A. baumannii* cell surface hydrophobicity on the capacity to form pellicle. While Nait Chabane et al. (2014) and Giles et al. (2015) observed a strong link between hydrophobicity and pellicle formation, McQueary and Actis (2011) found no correlation between these phenotypes. Our data show that while hydrophobicity might present a favorable property for pellicle formation, other cellular features should be involved, since none of the IC II strains with hydrophobic character demonstrated pellicle phenotype. However, hydrophobicity was clearly associated with the *A. baumannii* capacity to form conventional biofilm and adhere to the abiotic (plastic) surface, thereby suggesting that different surface properties underlie pellicle and conventional biofilm phenotypes. The recent structural analysis of principal adhesin of Csu pili, involved in *A. baumannii* biofilm formation, revealed three hydrophobic finger-like loops, found to mediate attachment to the hydrophobic surface (Pakharukova et al., 2018). Whether Csu adhesin plays a similar role in the formation of the pellicle, shown to contain the *A. bumannii* pili subunits in the matrix (Nait Chabane et al., 2014), remains to be elucidated.

Whereas our study confirms a link between *A. baumannii* hydrophobicity and biofilm formation as well as adherence to the abiotic surface, it demonstrates that hydrophobic phenotype renders *A. baumannii* to become more sensitive to desiccation and weakens its ability to adhere to the epithelial cells. Moreover, we observed that *A. baumannii* IC II strains, which displayed hydrophilic character, were more virulent compared to their hydrophobic counterparts using both *C. elegans* and murine infection models. This is in accordance with the observations made by Kempf et al. (2012) from the analysis of two *A. baumannii* strains recovered from the same patient, where a strain with hydrophobic features and biofilm forming ability did not show increased virulence compared to the strain with hydrophilic properties. Hu et al. (2016) provided data showing that biofilm forming phenotype of IC II strains did not correlated with their epidemicity suggesting that other virulence factors contribute to the success of this global clone. Nevertheless, most of our tested *A. baumannii*, including outbreak strains formed biofilm, whereas IC II strains with hydrophilic character, increased desiccation resistance and adherence to the epithelium cells, were mostly sporadic. However, the listed features of hydrophilic *A. baumannii* strains might be superior at certain conditions such as long periods dryness or at the onset of host colonization and such strains might pose a high infection risk. The origin of significant diversification of cell surface hydrophobicity phenotype among closely related IC II strains, observed in this study, is of particular interest, since neither CPS synthesis locus type, nor OC locus type were found to be responsible for these phenotypic variations. It is possible that unknown point mutations or genetic rearrangements in so far identified types of K locus or OC locus could impair cell surface hydrophobicity, therefore comprehensive studies based on the deep-sequencing would be required. Moreover, changes in CPS or LOS resulting from the modifications could also play a role in cell surface hydrophobicity. It was observed, that phosphoethanolamine modification of lipid A reduces the fitness and decreases the biofilm formation in *A. baumannii* (Pelletier et al., 2013; Da Silva and Domingues, 2017). Strains with this type of lipid A modification, tend to demonstrate increased resistance to colistin. However, all our tested IC II isolates were found to be colistin-sensitive according to the analysis of minimal inhibitory concentration (MIC) (Supplementary Figure S3), suggesting that this modification is not responsible for the phenotypic differences. Possibly, unknown changes in CPS or LOS could play a role in cell surface hydrophobicity between hydrophilic IC II strains.

Tipton et al. (2015) have recently identified *A. baumannii* AB5075 strain generating two subpopulations with different virulence features. Virulent cells possessed a thicker capsule, showed increased resistance to hospital disinfectants and desiccation, reduced biofilm formation and were more virulent in animal models compared to the avirulent cells (Tipton et al., 2015; Chin et al., 2018). Chin et al. (2018) have shown the involvement of transcriptional regulator TetR in the phenotypic switch in *A. baumannii* AB5075 strain. Interestingly, the observed phenotypes for virulent subpopulation of *A. baumannii* AB5075 closely resemble the features of our investigated hydrophilic IC II strains, which were more virulent compared to the hydrophobic strains, therefore we have tested the *tetR* gene expression by qPCR in the representative strains II-a and II-f. However, no differences in gene expression were observed (data not shown), indicating additional adaptation pathways.

## Conclusion

Our study revealed a set of lineage-specific cell-surface-associated features of clinical *A. baumannii* strains belonging to the most spread clonal lineages, suggesting distinct adaptation strategies. Moreover, a significant diversification of cell surface hydrophobicity-related phenotypes and their association with the virulence at the intra-lineage level was demonstrated for strains belonging to international clone lineage II thereby implying the high pathogenicity potential of this expanding clone. Indeed several countries reported the replacement of IC I *A. baumannii* isolates by IC II (Kim et al., 2017; Pournaras et al., 2017). The observed variations in an armory of virulence-associated features among IC II strains might favor a particular life-style within the clinical environment resulting in the different spreading routes and interaction with the host.

## Author Contributions

JS and RK designed the experiments. JS, RK, CP, SK, and JA performed the experiments. JS and ES analyzed the data and wrote the manuscript. All of the authors critically reviewed the manuscript and approved the final version.

## Conflict of Interest Statement

The authors declare that the research was conducted in the absence of any commercial or financial relationships that could be construed as a potential conflict of interest.

## Footnotes

1 https://pubmlst.org/abaumannii/
