# Supplementary material for: Surface-Related Features and Virulence Among Acinetobacter baumannii Clinical Isolates Belonging to International Clones I and II
Source: Front Microbiol. 2019 Jan 8;9:3116. doi: 10.3389/fmicb.2018.03116 (PMC6331429; doi:10.3389/fmicb.2018.03116)
Supplement: Supplementary file 2 [file Table_2.pdf]

Supplementary Material

Surface-related features and virulence among *Acinetobacter baumannii* clinical isolates belonging to international clone I and II

Jūratė Skerniškytė[*], Renatas Krasauskas, Christine Péchoux, Saulius Kulakauskas, Julija Armalytė and Edita Sužiedėlienė

*** Correspondence:** Jūratė Skerniškytė, jurate.skerniskyte@gf.vu.lt

Supplementary Table S2. Oligonucleotides, plasmids and strains used in this study.

Primers	Sequence (5'to 3')	Product size (bp)	Purpose	Source
Eps1<br>Eps2	CATTAATAAGCGCAATATTGAC<br>GTTATATAAAGCGCTTGAGAAACCCTTTGTGTATTTATCT	1024	*wza* upstream region amplification	This study
Eps3<br>Eps4	TCTCAAGCGCTTTATATAAC<br>GTCGAGGCATTTCTGTCCTAGAAGCACCTTTGCTGTC	684	*wza* downstream region amplification	This study
Fjgms<br>Rjgms	AGGACAGAAATGCCTCGAC<br>ATCTCGGCTTGAACGAATT	854	*aac(3)-I* amplification	This study
EpgR	TGTATTCAGTTTTATATTCAGTATCAC		Conformation for *wza* deletion	This study
P-Ab-ITSF<br>P-Ab-ITSB	CATTATCACGGTAATTAGTG<br>AGAGCACTGTGCACTTAAG	208	*A. baumannii* specie detection	Chiang *et al.*, 2011

gnaaF<br>galuR	CNTAYTAYYTDACNCATAAAGC<br>GTCAACNACBGTDACCATTTC	various	K locus amplification and sequencing	This study
wzy11F<br>wzy11R	AACGTTGGGACTATAGCAACAAAT<br>CCTGTTTGATGGGGTGGTCT	841	*wzy11* detection/ KL2 identification	This study
KL27F<br>KL27R	GGAGACTGGCTGCAACCTTAT<br>AGAGCTGTGCACCCAAACAG	333	KL27 identification	This study
KL6F<br>KL6R	CCACTTGGGGGAGTGTGATT<br>AACTTTCCGCCCCCAACTTA	236	KL6 identification	This study
KL1F<br>KL1R	ACGAAAACAACAGTTATGGCGA<br>ATGGGGGAGGTGTTACAGGT	193	KL1 identification	This study
KL40F<br>KL40R	GGAAGTTAATCGTACTCTGCATTAT<br>ACACACCTGTAAGCTTGCCT	648	KL40 identification	This study
KL4F<br>KL4R	AACGCGGCAAACTTACTGGA<br>CGCCGATAAACCCACCTAAG	654	KL4 identification	This study
KL20F<br>KL20R	GAACGCGGCAAACTTACAGG<br>CAGGAGAGGGACCTGCAATC	934	KL20 identification	This study
RH1704<br>RH1705	CCCTACAAGGTCTTGCCAAT<br>CCTCAGCCCGTACTTACAAC	2116	OCL1 identification	Kenyon *et al.*, 2014, c
Citrato F1<br>Citrato R12	AATTTACAGTGGCACATTAGGTCCC<br>GCAGAGATACCAGCAGAGATACACG	722	*gltA* amplification and sequencing	*https://pubmlst.org*

gyrB_F<br>gyrB_R	TGAAGGCGGCTTATCTGAGT<br>GCTGGGTCTTTTTCCTGACA	594	*gyrB* amplification and sequencing	*https://pubmlst.org*
RA1<br>RA2	CCTGAATCTTCYGGTAAAAC<br>GTTTCTGGGCTGCCAAACATTAC	425	*recA* amplification and sequencing	*https://pubmlst.org*
gpi_F<br>gpi_R	GAAATTTCCGGAGCTCACAA<br>TCAGGAGCAATACCCCACTC	456	*gpi* amplification and sequencing	*https://pubmlst.org*
GDHB1F<br>GDHB775R	GCT ACT TTT ATG CAA CAG AGC C<br>GTTGAGTTGGCGTATGTTGTGC	774	*gdhB* amplification and sequencing	*https://pubmlst.org*
cpn60_F<br>cpn60_R	GGTGCTCAACTTGTTCGTGA<br>CACCGAAACCAGGAGCTTTA	640	*cpn60* amplification and sequencing	*https://pubmlst.org*
rpoD-F<br>rpoD-R	ACCCGTGAAGGTGAAATCAG<br>TTCAGCTGGAGCTTTAGCAAT	672	*rpoD* amplification and sequencing	*https://pubmlst.org*

**Plasmids**	**Relevant characteristics**	**Source**
pUC19_sacB	pUC19 derivative with *sacB* gene from *Bacillus sp.*	Laboratory collection
pUC19_sacB_UDwzaGm	pUC19_sacB derivative with upstream and downstream regions of *A. baumannii wza* gene and *aac(3)-I* gentamicin aminoglycoside cassette from clinical *A. baumanni* strain	This study

**Strains**	**Relevant characteristics**	**Source**
Escherichia coli OP50	Wild type, bacterial food source for *C. elegans*	Brenner, 1974
Acinetobacter baylyi ADP1	Wild type	ATCC 33305*

Acinetobacter baumannii III-a Δ*wza*	*wza* gene-negative mutant of *A. baumannii* strain III-a; markerless	This study

*ATCC, American Type Cuture Collection

Brenner, S. (1974). The genetics of *Caenorhabditis elegans*. *Genetic* 77, 71–94;

Chiang, M.C., Kuo, S.C., Chen, Y.C., Lee, Y.T., Chen, T.L., and Fung, C.P. (2011). Polymerase chain reaction assay for the detection of *Acinetobacter baumannii* in endotracheal aspirates from patients in the intensive care unit. *J Microbiol Immunol Infect.* 44(2), 106-10. doi: 10.1016/j.jmii.2010.04.003;

Kenyon, J.J., Nigro, S.J., and Hall, R.M. (2014). Variation in the OC Locus of *Acinetobacter baumannii* Genomes Predicts Extensive Structural Diversity in the Lipooligosaccharide. *PLoS ONE* 9(9):e107833. doi: 10.1371/journal.pone.0107833;

This publication made use of the *Acinetobacter baumannii* MLST website (https://pubmlst.org/ abaumannii/) sited at the University of Oxford (Jolley & Maiden 2010, BMC Bioinformatics, 11:595). The development of this site has been funded by the Wellcome Trust.
