# Supplementary material for: Surface-Related Features and Virulence Among Acinetobacter baumannii Clinical Isolates Belonging to International Clones I and II
Source: Front Microbiol. 2019 Jan 8;9:3116. doi: 10.3389/fmicb.2018.03116 (PMC6331429; doi:10.3389/fmicb.2018.03116)
Supplement: Supplementary file 3 [file Data_Sheet_1.PDF]

Supplementary Material

Surface-related features and virulence among *Acinetobacter baumannii* clinical isolates belonging to international clone I and II

Jūratė Skerniškytė*, Renatas Krasauskas, Christine Péchoux, Saulius Kulakauskas, Julija Armalytė and Edita Sužiedėlienė

*** Correspondence:** Jūratė Skerniškytė, jurate.skerniskyte@gf.vu.lt

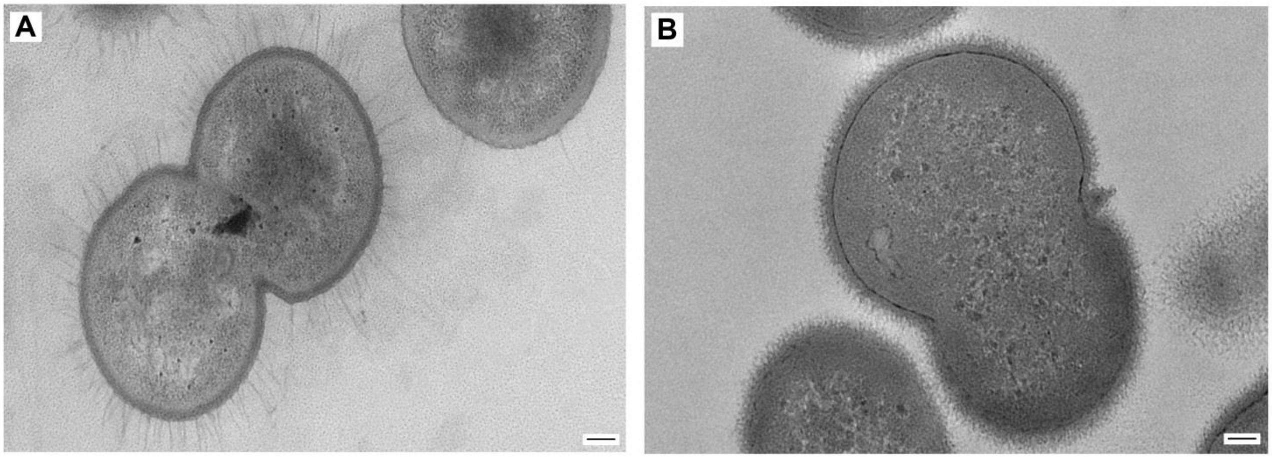

Supplementary Figure 1. Representative TEM images of *A. baumannii* IC I strain 169 (A) and IC II strain II-a (B). x15.000 magnification; scale bar is 70 nm.
