# Supplementary material for: Surface-Related Features and Virulence Among Acinetobacter baumannii Clinical Isolates Belonging to International Clones I and II
Source: Front Microbiol. 2019 Jan 8;9:3116. doi: 10.3389/fmicb.2018.03116 (PMC6331429; doi:10.3389/fmicb.2018.03116)
Supplement: Supplementary file 4 [file Data_Sheet_2.PDF]

Supplementary Material

Surface-related features and virulence among *Acinetobacter baumannii* clinical isolates belonging to international clone I and II

Jūratė Skerniškytė*, Renatas Krasauskas, Christine Péchoux, Saulius Kulakauskas, Julija Armalytė and Edita Sužiedėlienė

*** Correspondence:** Jūratė Skerniškytė, jurate.skerniskyte@gf.vu.lt

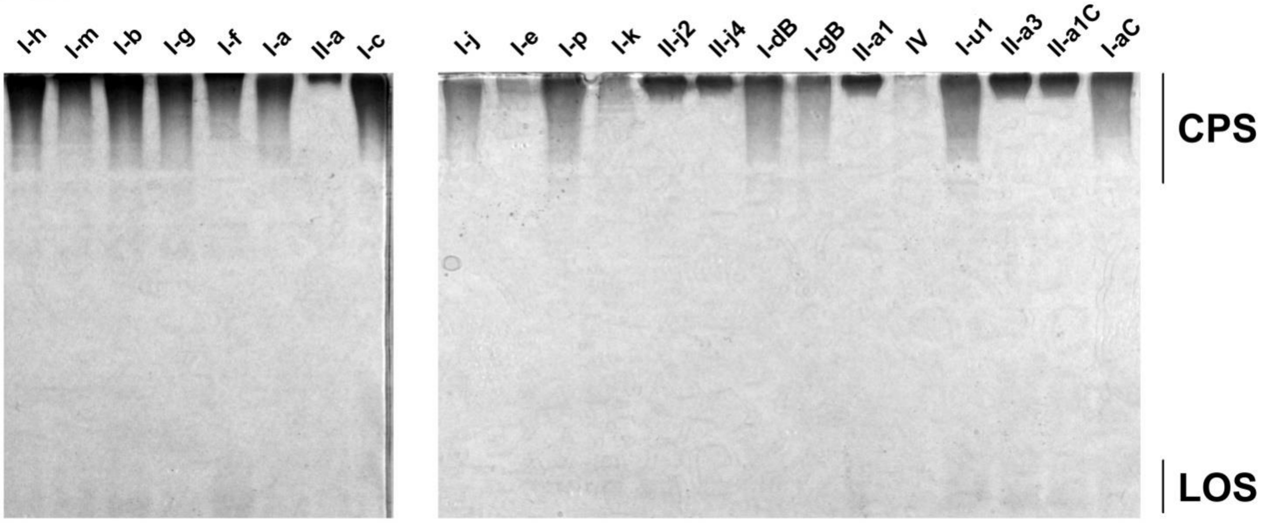

Supplementary Figure S2. Capsular polysaccharide (CPS) profiles of *A. baumannii* IC I and IC II lineage strains. 12% SDS-PAGE, followed by Alcian Blue staining was undertaken to visualize CPS. LOS denotes lipooligosaccharides. Roman numerals I and II in the strains names indicate IC I and IC II, respectively. IC I and IC II strains appear in the mixed order.
