# Supplementary material for: Surface-Related Features and Virulence Among Acinetobacter baumannii Clinical Isolates Belonging to International Clones I and II
Source: Front Microbiol. 2019 Jan 8;9:3116. doi: 10.3389/fmicb.2018.03116 (PMC6331429; doi:10.3389/fmicb.2018.03116)
Supplement: Supplementary file 5 [file Data_Sheet_3.PDF]

Supplementary Material

Surface-related features and virulence among *Acinetobacter baumannii* clinical isolates belonging to international clone I and II

Jūratė Skerniškytė[*], Renatas Krasauskas, Christine Péchoux, Saulius Kulakauskas, Julija Armalytė and Edita Sužiedėlienė

*** Correspondence:** Jūratė Skerniškytė, jurate.skerniskyte@gf.vu.lt

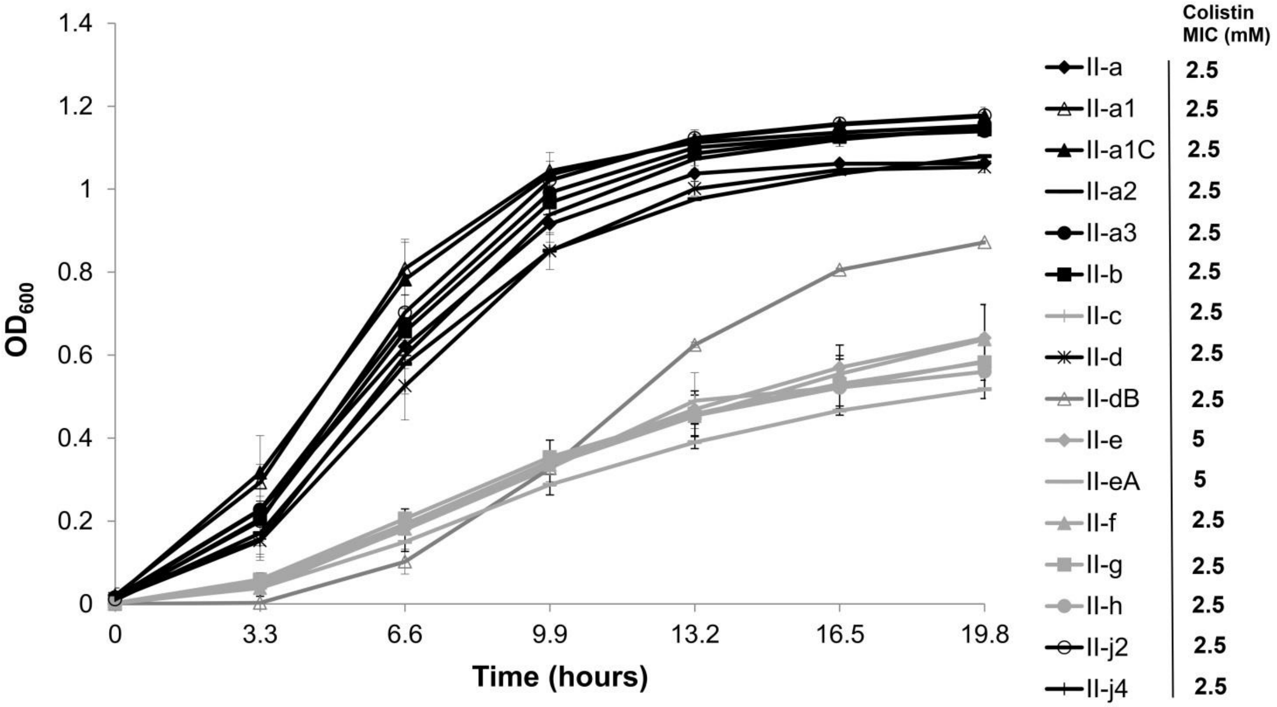

Supplementary Figure S3. Growth curves of IC II strains in LB medium. Grey curves indicate strains with reduced fitness. Error bars expressed as standard deviations from three independent experiments performed in triplicates. Colistin MICs of each strain are shown on the right.

Antimicrobial susceptibility testing

Colistin sulfate Minimal Inhibitory Concentrations (MICs) were determined using the broth microdilution method in duplicate according to the Clinical and Laboratory Standards Institute (CLSI) protocol (CLSI, 2013).

Clinical and Laboratory Standard Institute. Performance standards for antimicrobial susceptibility testing. (2013). Nineteenth informational supplement. CLSI document M100 S19. CLSI. Wayne. PA. USA.
