# Supplementary material for: Surface-Related Features and Virulence Among Acinetobacter baumannii Clinical Isolates Belonging to International Clones I and II
Source: Front Microbiol. 2019 Jan 8;9:3116. doi: 10.3389/fmicb.2018.03116 (PMC6331429; doi:10.3389/fmicb.2018.03116)
Supplement: Supplementary file 6 [file Data_Sheet_4.PDF]

Supplementary Material

Surface-related features and virulence among *Acinetobacter baumannii* clinical isolates belonging to international clone I and II

Jūratė Skerniškytė*, Renatas Krasauskas, Christine Péchoux, Saulius Kulakauskas, Julija Armalytė and Edita Sužiedėlienė

*** Correspondence:** Jūratė Skerniškytė, jurate.skerniskyte@gf.vu.lt

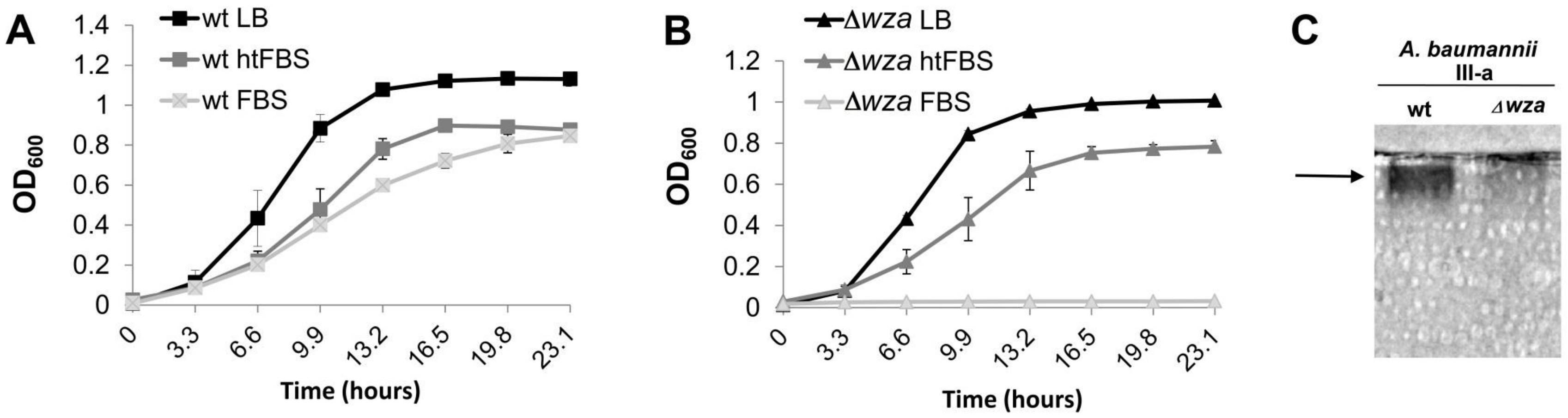

Supplementary Figure S4. Growth curves and CSP production by *A. baumannii* IC II strain III-a and its *wza* gene deletion mutant. A–B: growth curves of III-a wt (A) and Δ*wza* (B) strains in LB, LB supplemented with 80% of heat treated (htFBS) and untreated FBS; C – SDS-PAGE of polysaccharide extracts from III-a wt and Δ*wza* cells cultured for ~24 hours in LB; arrow indicates CPS fraction.
