# Supplementary material for: Surface-Related Features and Virulence Among Acinetobacter baumannii Clinical Isolates Belonging to International Clones I and II
Source: Front Microbiol. 2019 Jan 8;9:3116. doi: 10.3389/fmicb.2018.03116 (PMC6331429; doi:10.3389/fmicb.2018.03116)
Supplement: Supplementary file 7 [file Data_Sheet_5.PDF]

Supplementary Material

Surface-related features and virulence among *Acinetobacter baumannii* clinical isolates belonging to international clone I and II

Jūratė Skerniškytė*, Renatas Krasauskas, Christine Péchoux, Saulius Kulakauskas, Julija Armalytė and Edita Sužiedėlienė

*** Correspondence:** Jūratė Skerniškytė, jurate.skerniskyte@gf.vu.lt

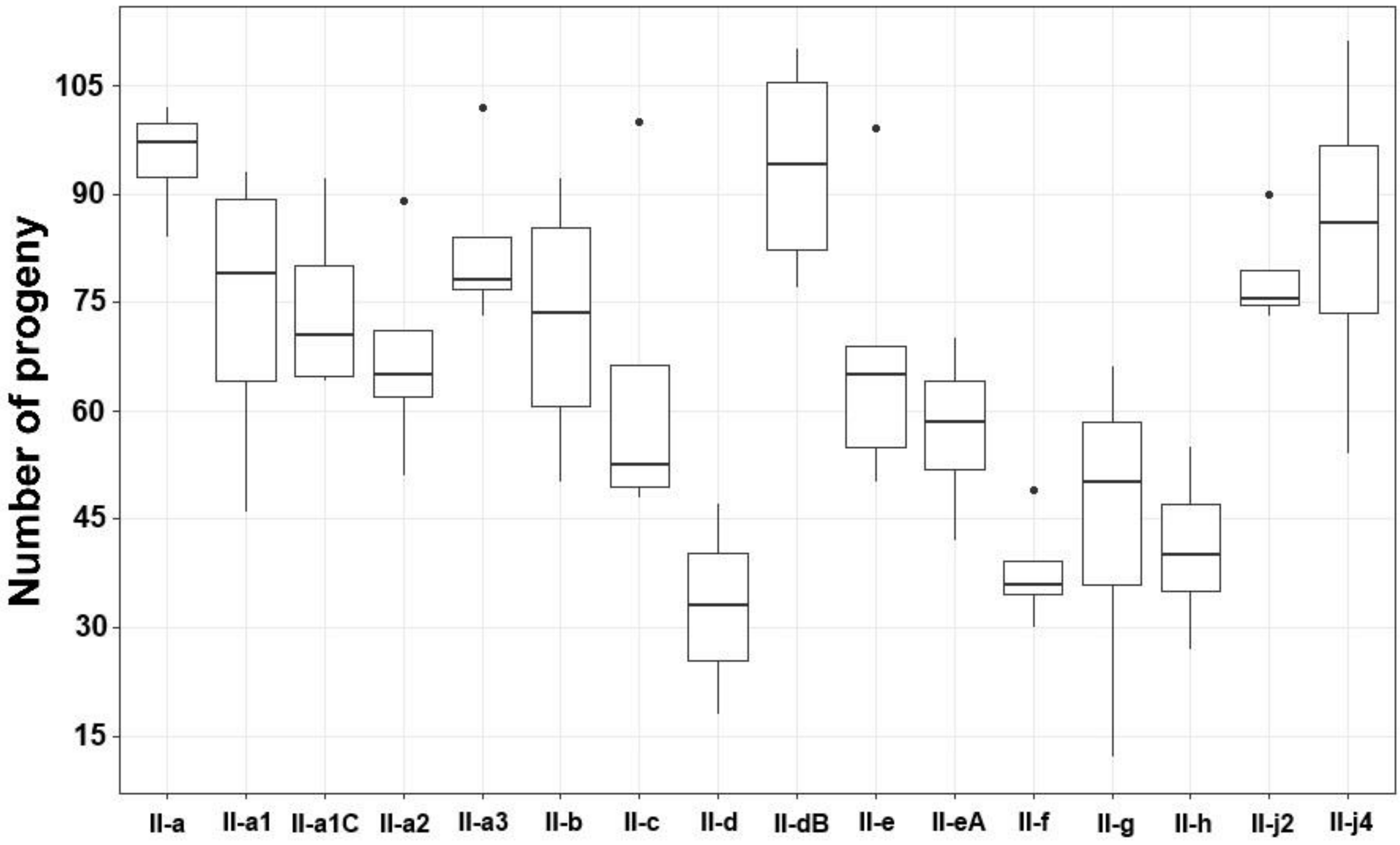

Supplementary Figure S5. *C. elegans* fertility assay. Box plot of the count of nematodes progeny after three days of incubation in the presence of *A. baumannii* IC II lineage strains exhibiting different cell surface hydrophobicity. Data are from at least three repeats. Black lines represent medians, whiskers – minimum to maximum values.
