# Supplementary material for: Surface-Related Features and Virulence Among Acinetobacter baumannii Clinical Isolates Belonging to International Clones I and II
Source: Front Microbiol. 2019 Jan 8;9:3116. doi: 10.3389/fmicb.2018.03116 (PMC6331429; doi:10.3389/fmicb.2018.03116)
Supplement: Supplementary file 8 [file Data_Sheet_6.PDF]

Supplementary Material

Surface-related features and virulence among *Acinetobacter baumannii* clinical isolates belonging to international clone I and II

Jūratė Skerniškytė[*], Renatas Krasauskas, Christine Péchoux, Saulius Kulakauskas, Julija Armalytė and Edita Sužiedėlienė

*** Correspondence:** Jūratė Skerniškytė, jurate.skerniskyte@gf.vu.lt

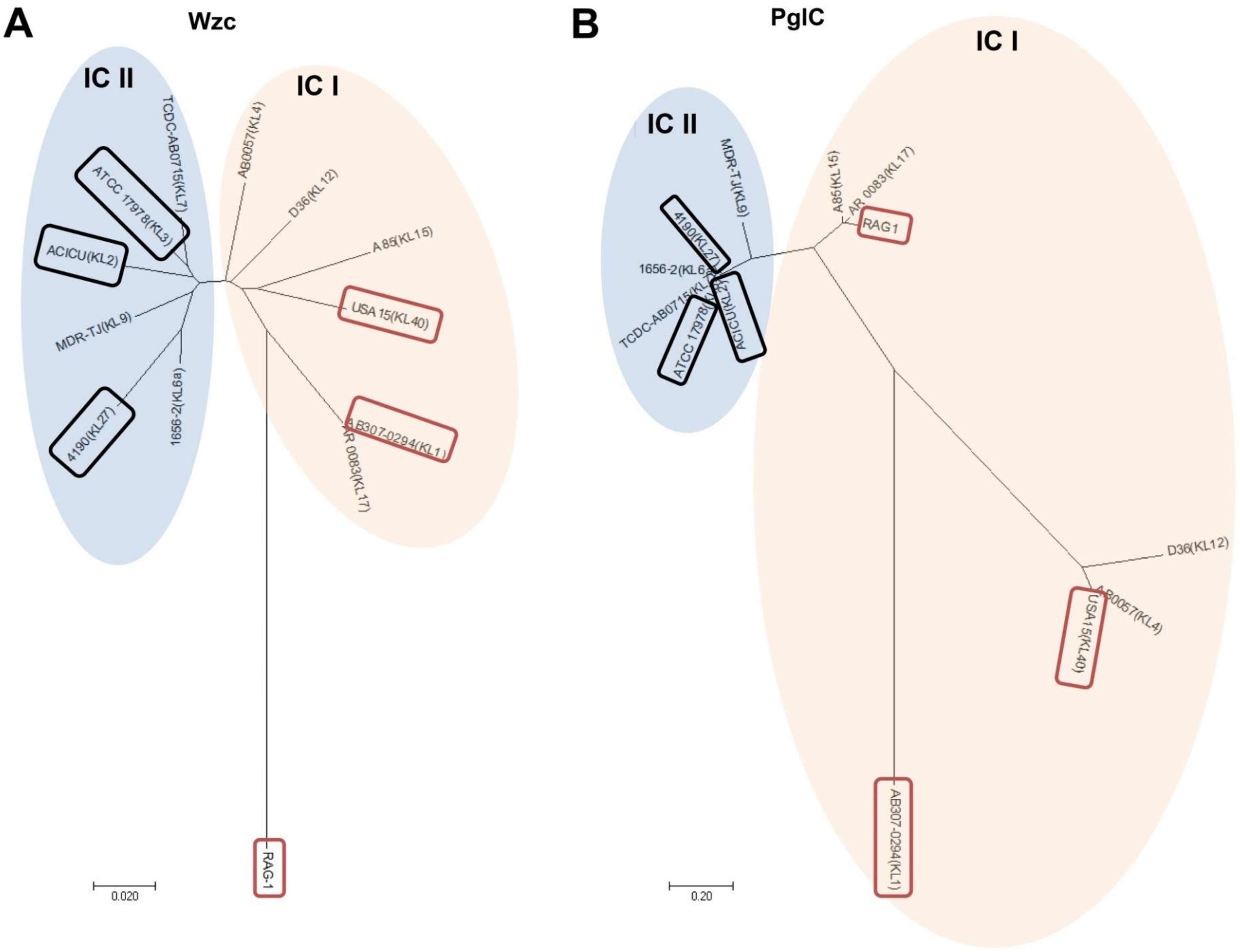
A
Wzc
IC II
IC I
TCDC-AB0715(KL7)
ATCC 17978(KL3)
ACICU(KL2)
MDR-TJ(KL9)
4190(KL27)
1656-2(KL6a)
AB0057(KL4)
D36(KL12)
A85(KL15)
USA 15(KL40)
AB307-0294(KL1)
AR 0083(KL17)
RAG-1
0.020
B
PglC
IC II
IC I
MDR-TJ(KL9)
4190(KL27)
1656-2(KL6a)
ACICU(KL2)
ATCC 17978
A85(KL15)
AR 0083(KL17)
RAG1
D36(KL12)
AB0057(KL4)
USA15(KL40)
AB307-0294(KL1)
0.20

Supplementary Figure S6. Phylogenetic analysis of *Acinetobacter* Wzc (A) and PglC (B) proteins. Orange and blue areas indicate strains belonging to IC I and IC II, respectively. *A. venetianus* strain Rag-1 and *A. baumannii* strains with different K locus (KL) types were included into analysis. Red and black squares indicate strains, for which CPS profiles have been determined as VM and HM, respectively. The scale bars indicate an evolutionary distance of amino acid substitutions per position in the sequence. Phylogenetic tree was generated using the Neighbor-Joining method by MEGA version 7.0 software.
